# Surface Mn(II) oxidation actuated by a multicopper oxidase in a soil bacterium leads to the formation of manganese oxide minerals

**DOI:** 10.1038/srep10895

**Published:** 2015-06-03

**Authors:** Zhen Zhang, Zhongming Zhang, Hong Chen, Jin Liu, Chang Liu, Hong Ni, Changsong Zhao, Muhammad Ali, Fan Liu, Lin Li

**Affiliations:** 1 State Key Laboratory of Agricultural Microbiology, Huazhong Agricultural University, Wuhan 430070, China; 2 Key Laboratory of Subtropical Agricultural Resource and Environment, Ministry of Agriculture, Huazhong Agricultural University, Wuhan 430070, China; 3 School of Life Science, Hubei University, Wuhan 430062, China

## Abstract

In this manuscript, we report that a bacterial multicopper oxidase (MCO266) catalyzes Mn(II) oxidation on the cell surface, resulting in the surface deposition of Mn(III) and Mn(IV) oxides and the gradual formation of bulky oxide aggregates. These aggregates serve as nucleation centers for the formation of Mn oxide micronodules and Mn-rich sediments. A soil-borne *Escherichia coli* with high Mn(II)-oxidizing activity formed Mn(III)/Mn(IV) oxide deposit layers and aggregates under laboratory culture conditions. We engineered MCO266 onto the cell surfaces of both an activity-negative recipient and wild-type strains. The results confirmed that MCO266 governs Mn(II) oxidation and initiates the formation of deposits and aggregates. By contrast, a cell-free substrate, heat-killed strains, and intracellularly expressed or purified MCO266 failed to catalyze Mn(II) oxidation. However, purified MCO266 exhibited Mn(II)-oxidizing activity when combined with cell outer membrane component (COMC) fractions *in vitro*. We demonstrated that Mn(II) oxidation and aggregate formation occurred through an oxygen-dependent biotic transformation process that requires a certain minimum Mn(II) concentration. We propose an approximate electron transfer pathway in which MCO266 transfers only one electron to convert Mn(II) to Mn(III) and then cooperates with other COMC electron transporters to transfer the other electron required to oxidize Mn(III) to Mn(IV).

Manganese (Mn) oxides are highly reactive minerals formed via Mn(II) oxidation. Mn oxides are associated with the morphology, migration, and transformation processes of many substances in the environment and play a crucial role in the biogeochemical cycle of many elements^1^. In soils, Mn oxide minerals are derived from both the chemical and biological oxidation of soluble Mn(II) to stable Mn(IV) oxides. Chemogenesis resulting from spontaneous thermodynamic processes slowly mineralizes Mn oxides through chemical oxidation, surface catalysis, and collochemical agglomeration. By contrast, the reaction rates associated with the biological processes (mediated by various enzymes) that oxidize Mn(II) to form different types of Mn oxide minerals are markedly higher than chemical processes^1^,^2^. In general, biological Mn oxide mineralization occurs in surficial soils because biogenic Mn(II) oxidation of soluble Mn(II) to Mn(IV) oxides is an aerobic process that requires O_2_^3^,^4^,^5^,^6^. Under anaerobic conditions, Mn(II) is favorably converted to certain other Mn compounds, such as rhodochrosite (MnCO_3_)^7^ and MnS^8^,^9^.

Various microorganisms, particularly bacteria and fungi, are considered the primary driving forces in the biological formation of Mn oxides. Previous studies of several Mn(II)-oxidizing bacteria from aquatic habitats, such as *Leptothrix discophora* SS-1^10^, *Pseudomonas putida* MnB1^11^, *P. putida* GB-1^12^, and *Bacillus* sp. SG-1^13^, have characterized Mn(II) oxidation as an enzymatically catalyzed biochemical process. The genes associated with Mn(II) oxidation in these strains have been identified as *mofA*, *mofB*, and *mofC* in SS-1^10^, the *ccm* operon containing five or more genes in MnB1^11^, and *mnxA*, *B*, *C*, *D*, *E*, *F*, and *G*, which constitute a cluster in an operon in SG-1^14^. However, only a few studies have investigated the Mn oxidation by bacteria in either surface soils^15^ or soils surrounding Fe–Mn nodules^16^. Furthermore, the genes associated with Mn oxidation in terrestrial soil-borne bacteria have not yet been cloned or characterized. The elucidation of the mechanisms underlying biogenic Mn oxidation and the biomineralization of manganese oxides in soils continues to be a research goal.

Interestingly, certain Mn(II)-oxidizing genes in marine bacteria, including *moxA* in *Pedomicrobium* sp. ACM3067^17^ and *mnxG* in *Bacillus* SG-1^14^, share significant sequence similarity with bacterial multicopper oxidase (MCO). MCO is a widespread enzyme capable of catalyzing the oxidation of many inorganic and organic substrates. Several studies have reported that the bacterial oxidation of Mn(II) to form Mn(IV) oxides is strongly influenced by different MCOs^18^,^19^. Bacterial MCOs are typically composed of three distinct cupredoxin domains. Only the C-terminal domain III containing a type I copper (Cu) site transfers electrons from the substrate to the trinuclear Cu center composed of a type II Cu atom and a pair of type III Cu atoms^20^. In *Bacillus* sp. strain PL-12 and *Bacillus* SG-1, the oxidation of Mn from soluble Mn(II) to Mn(IV) oxides has been described as a two-step reaction catalyzed by an MCO-containing complex, in which a single electron is transferred from Mn(II) to the type 1 Cu site and relayed to O_2_ at the type 3 Cu site^3^,^21^. Moreover, various bacterial MCOs have a wide range of substrate specificities, including aromatic amine and annular polyol (benzenediol), and the substrates may even include low-quasi-valence transition metals, such as Fe(II), Cu(I), and Mn(II)^22^. For example, an MCO from *Pseudomonas aeruginosa* is capable of oxidizing Fe(II) to Fe(III) after the acquisition of Fe(II) by the cell^23^. *Escherichia coli* CueO is also capable of oxidizing Cu(I) to Cu(II) using T1-type copper at the substrate-binding site and the trinuclear copper center at the oxygen-binding site^24^,^25^. A region rich in methionine near the T1 copper site that senses copper is required for the binding activity of the fifth copper^24^,^25^,^26^. These MCO-mediated oxidation reactions all appear to be related to the cell surface; thus, investigating the role of MCOs in Mn oxidation on the cell surface will improve the understanding of the biogenesis of Mn oxide minerals in the soil environment.

The objective of the current study was to define the role of an MCO protein (MCO266) from a soil-borne Mn(II)-oxidizing bacterium in bacterial cell-surface-orientated Mn oxidation and the biomineralization of Mn oxides. For this purpose, an MCO heterologous expression system for Mn(II) oxidation was established using the surface-display system of *E. coli*. Using this approach, we confirmed the biological formation of Mn oxides (III and IV) catalyzed by surface-expressed MCO266 proteins. Following continuous Mn enrichment in laboratory shake-flask trials, recombinant strains with MCO266 were able to catalyze the formation of Mn oxide deposits on the cell surface, resulting in the successive formation of bulky, regular, microspherical aggregates. Therefore, MCO266 is involved in the biological nucleation of Mn oxides under laboratory conditions.

## Results

### Characterization of Mn(II)-oxidizing *E. coli* MB266

**Figure 1:**
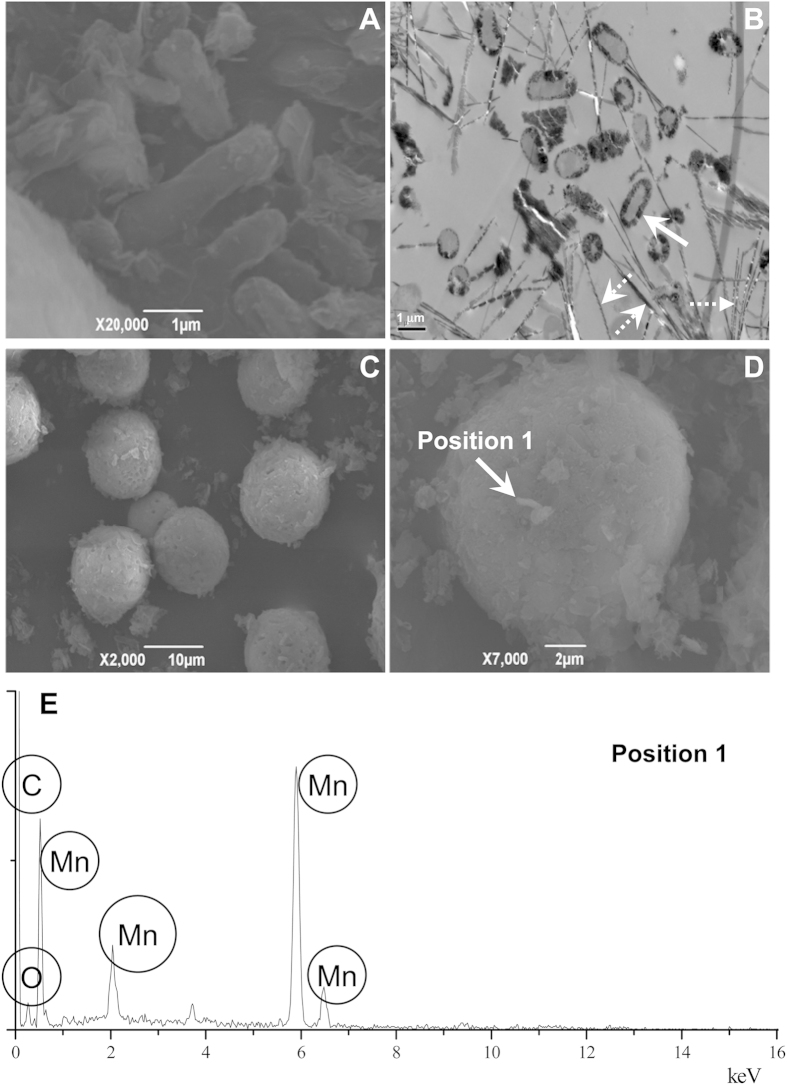
SEM and TEM micrographs of MB266 and EDX spectrogram. **A**: SEM micrograph of the dissociated MB266 and Mn oxide sediments; **B**: TEM micrograph of MB266 and Mn oxide deposits; **C**: SEM micrograph of the Mn oxide aggregates produced by MB266; **D**: SEM micrograph of the Mn oxide aggregates produced by MB266; **E**: EDX spectrogram of a randomly selected EDX assay position (indicated by the arrow in Fig. 1D).

We previously identified a soil-borne *E. coli* strain (named MB266) in a brown soil that surrounded Fe−Mn nodules^27^. Mn oxide formation was detectable in this strain after culturing in Lept medium for 48 h. Within 120 h, the concentration of Mn oxides [quantified using a standard Leucoberbelin Blue (LBB) spectrophotometry assay] reached 65 μM. Scanning electron microscopy (SEM) revealed that the surfaces of the MB266 cells exhibited rough and irregular deposit layers (Fig. 1A). Transmission electron microscopy (TEM) also showed a thick non-transparent deposit layer on the surface of MB266 cells in conjunction with dark-brown acicular deposits (Fig. 1B). This strain was subjected to continuous Mn(II) enrichment in a laboratory shake-flask trial for 14 d to investigate its micromorphological and physiological responses. Unexpectedly, regular microspherical aggregates with diameters of approximately 10 μm to 20 μm were observed via SEM, and the bacteria attached to and embedded in the aggregates were easily distinguished (Fig. 1C,D). Energy-dispersive X-ray (EDX) spectroscopic scanning assays revealed that the surfaces of the aggregates were mainly covered with Mn, in addition to the elements carbon (C) and oxygen (O) (Fig. 1E).

### Multicopper oxidase is involved in Mn oxidation

To identify the genes responsible for the Mn(II)-oxidizing activity of MB266, a mutant library of MB266 was constructed via the conjugal transfer of mini-Tn5 (Fig. S1). From the approximately 1,500 transconjugants, we obtained three candidate mutants with significantly reduced Mn(II)-oxidizing activities, as detected through LBB assays. Nucleotide sequencing and BLASTN analysis of the inverse polymerase chain reaction (PCR)-amplified fragment flanking the Tn5 insertion site from one mutant revealed that the insertion occurred in the multicopper oxidase (MCO266) gene locus. This gene (*mco266*) (GenBank accession number: JF682492) was amplified from the MB266 genome. The sequence analysis of *mco266* revealed that the putative MCO266 protein contained four typical Cu-binding sites located in regions surrounding the aa residues H_101_−H_103_, H_141_−H_143_, H_443_−H_448_, and H_499_−H_505_ in its intermolecular structure. The molecular organization of the putative MCO266 revealed that the N-terminal moiety contains one type 2 and two type 3 copper-binding sites coordinated by eight histidine residues. By contrast, the C-terminal moiety contains a type 1 copper-binding site consisting of two histidine residues and one cysteine residue, as well as a methionine-rich region. Although MCO266 showed an aa sequence similarity of less than 20% compared with several other well-known Mn(II)-oxidizing proteins (e.g., MnxG and MofA), two conserved copper-oxidizing superfamily domains and two conserved Cu(II)-binding sites corresponding to domains in the MnxG structure were identified in this protein. Thus, we selected *mco266* as a target gene for further study.

**Figure 2:**
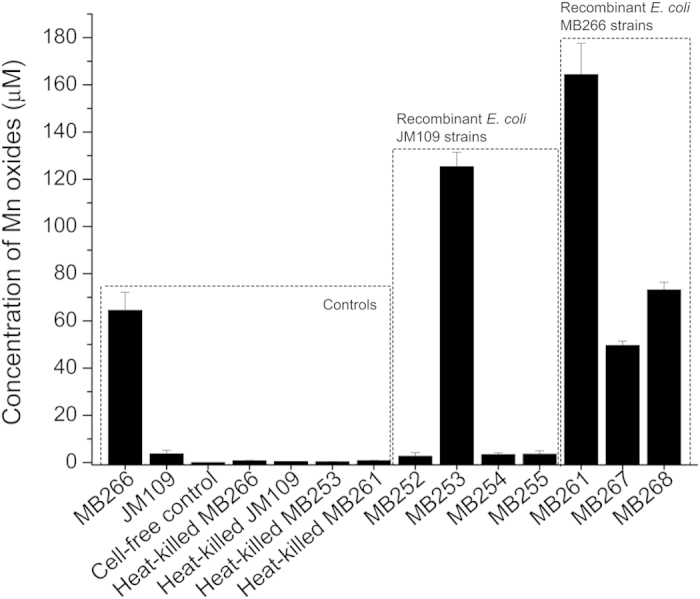
Mn(II)-oxidizing activity measurement of the recombinant *E. coli* strains. All of the strains (Table S3) were grown in liquid Lept medium in the presence of 1 mM Mn(II) for 5 d. The Mn oxide concentrations produced by these strains were then measured using the LBB method. For comparison purposes, a cell-free liquid Lept medium, heat-killed *E. coli* MB266, JM109, MB253 and MB261, wild-type MB266 and the recipient strain JM109 were subjected to the same measurement protocol in parallel.

**Figure 3:**
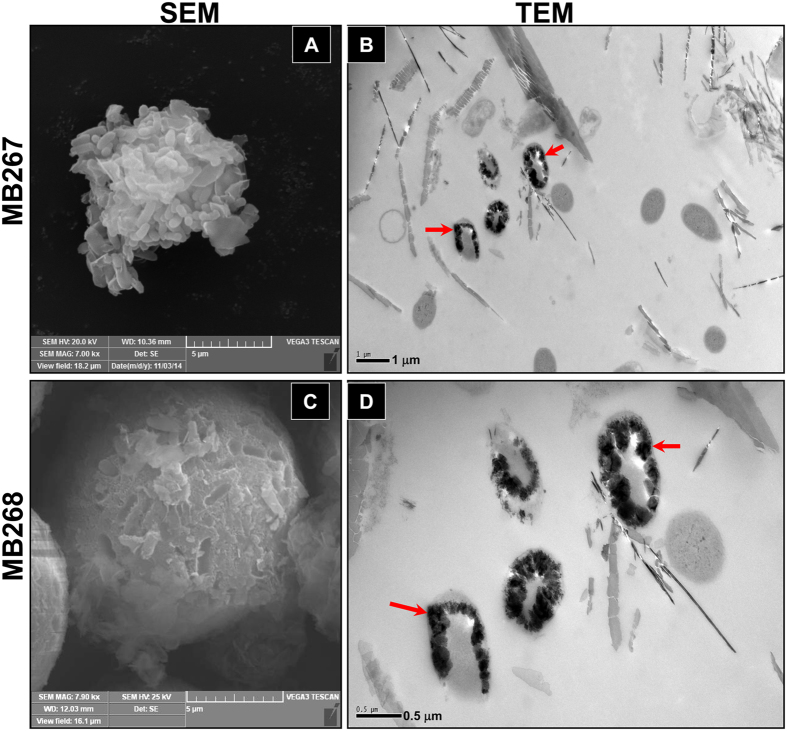
SEM and TEM micrographs of the *mco266*-disrupted mutant MB267 and recombinant MB268 compensated with MCO266. **A** and **C**: Aggregates formed by MB267 and MB268, respectively. **B** and **D**: Mn oxide deposit layers produced by MB267 and MB268 cells, respectively.

A mutant strain of *E. coli* MB266 with a site-specific gene disruption of *mco266* was developed using the Red recombination strategy^28^. The Mn(II)-oxidizing activity of the *mco266*-disrupted mutant strain MB267 was observed to be approximately 37% lower than that of MB266 (Fig. 2). The plasmid-mediated intracellular *mco266* expression in MB267 completely compensated for the reduced activity, and the resultant *E. coli* MB268 strain exhibited even higher activity than MB266 (Fig. 2). However, after introducing the *mco266* gene into the activity-negative recipient strain *E. coli* JM109, the resultant MB252 strain failed to exhibit any Mn(II)-oxidizing activity (Fig. 2). Surprisingly, SEM images revealed that the mutant strain MB267 formed irregular and loose cell aggregates after continuous Mn^2+^-enriched culturing for 14 d (Fig. 3A). This result differs significantly from the regular and compact microspherical aggregates composed of rare bacterial cells formed by MB268 (Fig. 3B) and wild-type MB266 (Fig. 1D), although similar rough and non-transparent deposit layers were observed in association with MB267 cells (Fig. 3B) and MB268 cells (Fig. 3D). These results indicate that the MCO266 protein from the strain MB266 is involved in Mn(II) oxidation.

### Surface display of MCO266 and Mn(II)-oxidizing activity of intact cells and cell fractions

Next, we verified the surface occurrence of MCO266-mediated Mn(II) oxidation. We performed cell-surface-display experiments to immobilize the MCO266 protein on the surfaces of both *E. coli* JM109 and MB266 cells using the N-terminal domain of an ice nucleation protein (InaQ)^29^ from *Pseudomonas syringae* as the anchor. Immunomicroscopic examination (Fig. S2A), Western blot analysis of the cell fractions (Fig. S2B), and fluorescence-activated cell sorting (Fig. S2C) of the resultant *E. coli* MB253 and *E. coli* MB261 cells confirmed the surface localization of the fusion protein InaQ-N/MCO266 in both strains.

The whole-cell Mn(II)-oxidizing activities of all recombinant *E. coli* strains, the wild-type strain *E. coli* MB266 and the recipient strain *E. coli* JM109 were assayed under comparably normalized conditions. As shown in Fig. 2, MB266 retained an activity level comparable to that observed in its initial state^27^. By contrast, a cell-free substrate control, JM109, and heat-killed MB266, JM109, MB253 and MB261 exhibited nearly zero activity. However, the MB253 cells expressing surface-displayed MCO266 proteins exhibited very high activity, strongly contrasted with the activity-negative parent strain JM109 and the MB252 cells expressing intracellular MCO266. Significantly increased activity was also exhibited by recombinant MB261 cells expressing surface-displayed MCO266, and these cells exhibited higher activity than the parent strain MB266. These results confirm the biogenesis of Mn oxidation and further demonstrate that MCO266 is involved in Mn(II) oxidation on the cell surface.

Samples of the purified MCO266 protein and the cell outer membrane component (COMC) fractions of MB266 and JM109 (separately or combined and with or without proteinase K proteolysis) were obtained to investigate their Mn(II)-oxidizing activities. In contrast to the limited background activity of the JM109 COMC fraction alone, the MB266 COMC fraction exhibited substantial activity (Fig. S3), further verifying the surface occurrence of Mn(II) oxidation in this strain. Notably, the purified MCO266 protein alone failed to exhibit the expected activity, but the level of activity was significantly higher in a mixture of purified MCO266 and the MB266 COMC fraction and/or the activity-negative JM109 COMC fraction (Fig. S3). However, the treatment of these fractions with proteinase K significantly reduced the joint Mn(II)-oxidizing activity of MCO266 with either the JM109 COMC or MB266 COMC fractions to background levels (Fig. S3, columns titled “MCO266+[JM109COMC+K]” and “MCO266+[MB266COMC+K]”). The proteinase K treatment also had a significant activity-reducing effect on the MB266 COMC fraction. These results suggest that MCO266-mediated Mn oxidation is associated with certain COMCs that appear to contain proteins. To determine the functional domains responsible for Mn(II) oxidation in the MCO266 molecules, the surfaces of *E. coli* JM109 cells were forced to express either the truncated N-terminal (MCO266-N, from aa 1 to aa 333) or the C-terminal moiety (MCO266-C, from aa 334 to aa 516) of MCO266 using the same anchor protein. Neither the resultant *E. coli* MB254 (displaying InaQ-N/MCO266-N) nor MB255 cells (displaying InaQ-N/MCO266-C) exhibited Mn(II)-oxidizing activity (Fig. 2), which indicates that the full-length MCO266 is required for Mn(II) oxidation.

### Microspherical aggregates of Mn oxides formed by recombinant *E. coli* MB261 and MB253 cells

**Figure 4:**
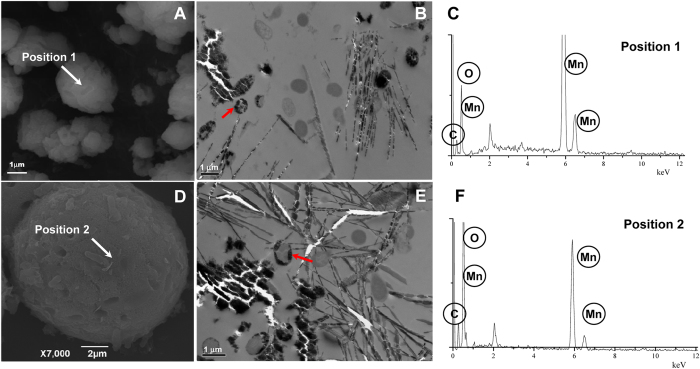
SEM and TEM micrographs and EDX spectrograms of the recombinant *E. coli* strains MB253 and MB261. **A** and **D**: SEM micrographs of the aggregates produced by MB253 and MB261, respectively; **B** and **E**: TEM micrographs of the Mn oxide deposits produced by MB253 and MB261, respectively; **C** and **F**: EDX spectrograms of randomly selected EDX assay positions, which are indicated by the arrows in **A** and **D** respectively.

Recombinant *E. coli* MB253 and MB261 cells with surface-displayed MCO266 proteins were subjected to continuous 14-d cultivation with 1 mM Mn(II). Not unexpectedly, SEM images showed that microspherical aggregates were formed by both MB253 and MB261 cultures (Fig. 4A,D). The regular shape and diameter of these MB261 aggregates were very similar to those of the MB266 aggregates (Fig. 1C,D). Conversely, the MB253 aggregates were irregular in shape and smaller (<5 μm in diameter) compared with those of MB261. The MB253 and MB261 cells were wrapped with rough and non-transparent deposit layers (Fig. 4B,D). The TEM images showed brown acicular deposits surrounding the MB253 and MB261 cells, consistent with the observations from the MB266 cells (Fig. 1B). EDX scanning of randomly selected sites on the microspherical aggregate surfaces revealed that Mn was the dominant element, but other elements, including C and O, were also present (Fig. 4B, position 1 for the MB253 aggregate; 4D, position 2 for the MB261 aggregate).

### Kinetic analysis of Mn oxidation and aggregation formation by MB253 and MB261

**Figure 5:**
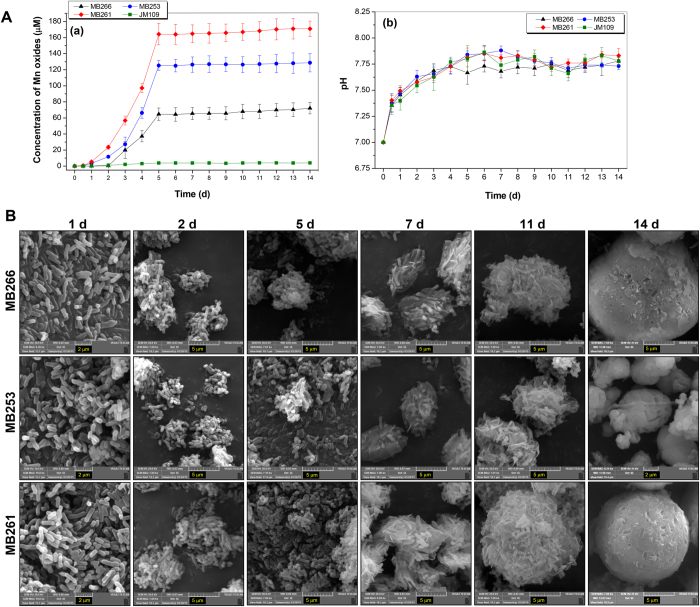
Kinetic analysis of (A) Mn(II) oxidation and (B) aggregate formation by the wild-type strain MB266 and recombinant strains MB253 and MB261 expressing surface-located MCO266. **A**: The Mn(II)-oxidizing activities (**a**) and pH (**b**) were measured over a period of 14 d during which the cells were grown in liquid Lept medium in the presence of 1 mM Mn(II). **B**: The cultures were examined via SEM on days 1, 2, 5, 7, 11, and 14.

Due to their apparent high Mn(II)-oxidizing activity and their ability to catalyze aggregate formation, MB253 and MB261 cells were selected as the target strains for a kinetic examination using a 14-d culturing period with an initial Mn(II) concentration of 1 mM. The whole-cell Mn(II)-oxidizing activities of MB253 and MB261 steadily increased over the first 5 d to the maximum observed values and maintained these activities throughout the experiment (Fig. 5Aa). Thus, the pattern of these cells was synchronous with that of the wild-type strain MB266. The pH levels of all the culture suspensions were maintained at 7.3 to 7.8 (Fig. 5Ab) throughout the initial 5-d period and reached a stable level of <8.0 over the course of the 14-d culturing process. Therefore, these results demonstrate that these strains exhibited biological Mn-oxidation activity. SEM images (Fig. 5B) showed that the aggregate formation processes in MB266, MB253 and MB261 were similar, consistent with their Mn(II)-oxidizing activities during the culturing period. In addition to the production and accumulation of Mn oxides, the bacterial cells gradually gathered during days 2 to 5, formed loose aggregates during days 7 to 11, and ultimately formed more compact microspherical aggregates on day 14. TEM images (Fig. S4) also captured the gradual formation of deposit layers and acicular deposits from zero deposition (day 1) to deposit spots (day 2), deposit layers (days 5 to 7), and layers and acicular deposits (days 11 to 14) during the culturing of all three strains.

### XPS and XRD characterization of Mn oxides formed by MB261 and MB253 cells

**Figure 6:**
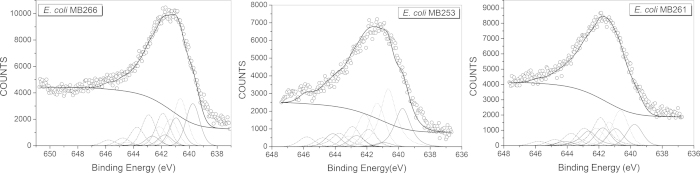
XPS patterns of the Mn (2p3/2) spectrogram of the Mn oxides produced by the *E. coli* MB266, MB253, and MB261 cultures. The upper circles represent the observed data. The thick, solid curve indicates the best fit of the data. The dashed–dotted curves represent the Mn(IV) multiplet peaks, the dotted lines represent Mn(III), and the thin solid lines represent Mn(II).

X-ray photoelectron spectroscopy (XPS) analysis was performed to calculate the relative quantities of Mn(III), Mn(IV) oxides and Mn(II) near the surface relative to the total quantities of Mn oxides produced by the *E. coli* MB261, MB253 and MB266 cultures. Figure 6 shows the XPS narrow scans of Mn (2p_3/2_) for the Mn oxides formed by MB266, MB261, and MB253. The Mn (2p_3/2_) spectra demonstrated the existence of Mn(IV) and Mn(III) oxides, as well as Mn(II), in the biotic Mn oxides. The binding energies, relative intensities of all of the peaks in each multiplet set, and the full width at half maximum were determined and are presented in Table S1. The proportions of Mn(IV)-oxides, Mn(III)-oxides and Mn(II) in the total quantity of Mn oxides formed by MB266 were found to be 33.68%, 34.02%, and 32.29%, respectively; those of MB261 were 31.74%, 34.73%, and 33.51%, respectively; and those of MB253 were 24.57%, 52.8%, and 22.63%, respectively.

**Figure 7:**
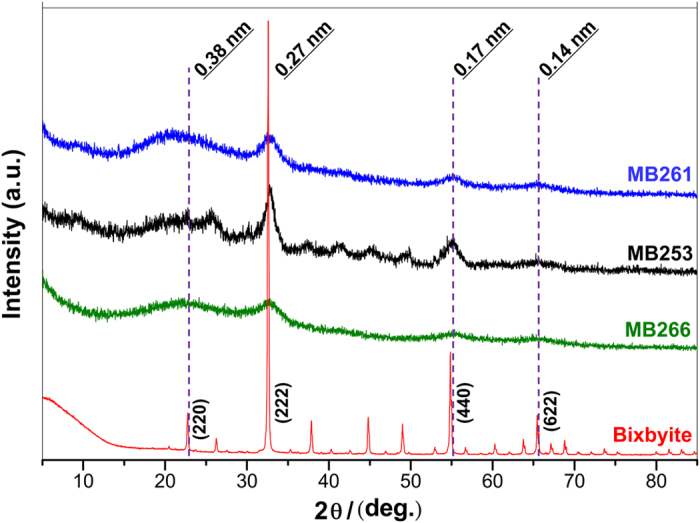
XRD patterns of Mn oxides produced by the recombinant *E. coli* MB261 and MB253 strains and the wild-type MB266 strain. The experiments were performed using dried powdered Mn oxide samples. The dashed lines indicate the peaks that overlap with standard bixbyite (Mn_2_O_3_).

X-ray diffraction (XRD) assays were also conducted to verify the types of Mn oxides in the microspherical aggregates formed by MB261, MB253 and MB266 cells. As shown in Fig. 7, the power XRD profile of the natural pure bixbyite exhibits sharp peaks at positions corresponding to those of Mn_2_O_3_ (JCPDS 02-0896). As expected, the XRD patterns of the Mn oxides from the microspherical aggregates from all three strains exhibit four distinct peaks at 0.38, 0.27, 0.17, and 0.14 nm, which correspond to the (220), (222), (440) and (622) crystal planes, respectively. These results indicate the presence of Mn_2_O_3_ in all three strains’ microspherical aggregate samples. However, all the strains’ Mn oxide peaks were relatively low in intensity and exhibited relatively broad areas compared with those of bixbyite; thus, the biogenic Mn oxides may be poorly crystallized bixbyite.

### Effect of Mn(II) concentrations and different chemicals on Mn(II)-oxidizing activity and aggregate formation

To facilitate the assays, the strain MB253 derived from the activity-negative recipient strain JM109 was used to investigate the effects of Mn(II) concentrations and different chemicals on Mn(II)-oxidizing activity and aggregate formation. As shown in Fig. S5A, as the initial Mn(II) concentrations increased from 0.1 mM to 2.0 mM, both MB253 and MB266 exhibited steadily increasing Mn(II)-oxidizing activities. By contrast, at 0.05 Mn(II), no activity was detected in either strain. SEM micrographs showed that both MB266 (Fig. S5B, a) and MB253 (Fig. S5B, e) formed regular and compact microspherical aggregates following incubation with 1 mM Mn(II). By contrast, incubations with 0.5 mM Mn(II) (Fig. S5B, b for MB266; Fig. S5B, f for MB253) and 0.2 mM Mn(II) (Fig. S5B, c for MB266; Fig. S5B, g for MB253) resulted in irregular and incompact aggregates, and an incubation with 0.1 mM Mn(II) produced no observable aggregates (Fig. S5B, d for MB266; Fig. S5B, h for MB253). These results confirm that Mn(II) oxidation does not idiomorphically occur in these strains but requires the concentration of Mn(II) to exceed certain thresholds. Furthermore, because the process is synchronous with Mn(II) oxidation, the formation of aggregates requires the production of a certain quantity of Mn oxides by these strains.

The effects of various chemical compounds on the Mn(II)-oxidizing activities of MB253 and MB266 were investigated. Table S2 shows that a well-known inhibitor of MCOs^30^, sodium azide, significantly inhibited MB253 activity (approximately 7% and 2% residual activities following treatment with 1 mM and 10 mM sodium azide, respectively), suggesting that MCO266 is the primary contributor to the Mn(II)-oxidizing activity of this strain. However, in MB266, the activity was higher (30% and 18%, following treatment with 1 mM and 10 mM sodium azide, respectively). This result suggests that this strain possesses one or more Mn(II)-oxidizing enzyme(s) in addition to MCO266. Moreover, higher concentrations of SDS (10 mM), cytochrome C (100 μM) and ATP (2000 μM) exhibited certain inhibitory effects on the Mn(II)-oxidizing activities of both strains, whereas three coenzymes (PQQ, NADH and NAD^+^) increased the Mn(II)-oxidizing activities of both strains. Thus, Mn oxidation is potentially associated with the energy metabolic pathways in the host cells.

## Discussion

This study demonstrates that MCO266 is an Mn(II)-oxidizing protein that actuates Mn oxide deposition onto the bacterial cell surface and successively forms oxide aggregates. Both wild-type *E. coli* MB266 and the recombinant *E. coli* strains with surface-displayed MCO266 are capable of producing microspherical aggregates of Mn oxides. Thus, soil-borne Mn(II)-oxidizing bacteria have the potential to serve as rudimentary or preliminary nucleating centers for the formation of ferromanganous nodules in natural soils. Various metallic/nonmetallic ions are localized in these centers and are adsorbed to the bacterial aggregates due to the strong oxidation and adsorption activities of the Mn oxides. Thus, the aggregates can ultimately initiate the formation of ferromanganese nodules and/or Mn oxide minerals in soil following polyfunctional interactions of various microorganisms and their chemical reactions with various elements^31^,^32^,^33^.

Although many studies have investigated the molecular mechanism of Mn oxidation in marine bacteria, soil-borne bacteria have rarely been studied. To identify the Mn(II)-oxidizing gene in *E. coli* MB266 that results in high Mn(II) oxidation activity, we constructed a transposon Tn5-insertion mutant library for MB266. The *mco266* gene located in the genomic sequences flanking a Tn5 insertion site manifested a positive response. Therefore, the Mn(II) oxidation activity of the strain MB266 is closely linked to the *mco266* gene. Sequence analysis revealed that *mco266* is a monocistronic gene, whereas the *mco266* genes of marine bacteria are located in polycistronic operons^10^,^12^,^14^. Because different gene organizations may lead to significant discrepancies between aqueous-phase and soil-borne bacteria in terms of the mechanisms of Mn(II) oxidation, an intensive study of MCO266 via heterologous expression and *in vitro* characterization of Mn(II)-oxidizing activity was performed. Significant catalytic activity was detected when MCO266 was mixed with the COMCs or immobilized on the surfaces of the target cells. Moreover, neither purified nor intracellularly expressed MCO266 was capable of Mn(II) oxidation. The results of *mco266* disruption and compensation *in trans* showed an obvious decrease in Mn(II)-oxidizing activity; however, the activity was not completely eradicated. Although the results demonstrated in principle that MCO266-actuated Mn oxidation is a surface-engendered event, other oxidative routes may also exist as a result of the adaptation of the cells to varied and inconstant soil environments.

In the natural environment, both abiotic and biological oxidation of Mn(II) to Mn(III/IV) may occur simultaneously; however, the abiotic oxidation of Mn(II) is known to occur preferentially at pH values greater than 8.0^34^,^35^. In the current study, the Mn(II) oxidation associated with several target strains was kinetically examined, and substantial Mn(II)-oxidizing activities were observed during the experiments at pH values less than 8.0 (Fig. 5Ab). Moreover, the cell-free substrate and all heat-killed strains failed to exhibit Mn(II)-oxidizing activity (Fig. 2). Therefore, Mn oxidation apparently occurs through a biotic transformation process. Furthermore, bacterial Mn oxidation has been found to be an aerobic biotransformation process^4^. For example, the Mn oxidation rate in *L. discophora* SS1 is directly proportional to the O_2_ concentration^6^, and Mn(II) failed to be converted into Mn(III/IV) in *L. discophora* SS1^6^ and *P. putida* GB-1^36^ under anaerobic conditions. To investigate whether MCO266-mediated Mn oxidation is also dependent on oxygen, we performed comparative measurements of the Mn(II)-oxidizing activity of both the wild-type stain MB266 and the engineered strains with surface-displayed MCO under aerobic and anaerobic conditions in the presence of 1 mM Mn(II). Interestingly, although *E. coli* strains are well-known facultative anaerobes, all of the tested strains exhibited very poor cell growth under the anaerobic conditions, and no Mn oxide could be detected, verifying that Mn oxidation by MCO266 requires O_2_ and that the presence of 1 mM Mn(II) may act as a restraining factor for cell growth in the absence of O_2_. Conceivably, the oxidation of Mn(II) to Mn oxide by MCO266 occurs through electron transfer to O_2_, which acts as the final electron receptor, as suggested by the results from *in vitro* experiments. These experiments demonstrated that the mixture of purified MCO266 and COMC complex exhibited Mn(II)-oxidizing activity, but the purified MCO266 alone did not (Fig. S3).

The SEM and TEM images obtained in this study revealed that the Mn oxide layers were located in close proximity to MB266 and the recombinant strains with surface-displayed MCO266 (Figs. 1,3, 4, 5). This result further supports the conclusion that the Mn(II) oxidation process occurs on the cell surface. Moreover, the surface location of MCO266 conferred activity to intrinsically inactive *E. coli* JM109. The increased activity observed in MB266 strongly suggests the existence of interactions between MCO266 and cell-surface components. The requirement for COMCs from either JM109 or MB266 for Mn(II) oxidation by purified MCO266 further confirms this interaction (Fig. S3). In general, MCO266-mediated oxidation is initiated by the receipt of an electron from substrates via type I Cu bound to MCO266-C, and the subsequent electron transfer to other substrates is conducted via three additional copper atoms bound to MCO266-N^37^. Thus, the cell-surface Mn(II) oxidation process mediated by MCO266 in the MB266 strain is most likely associated with the electron transfer process.

Mn oxidation from Mn(II) to Mn(III) or Mn(IV) by electron transfer from a series of redoxases has been previously described in other bacteria. Webb *et al*.^38^ revealed that Mn(II) oxidation by *Bacillus* SG-1 is mediated by two consecutive single-electron transfer reactions that require MnxG. In this process, Mn(III) is an instantaneous intermediate product, and the oxidation process from Mn(II) to Mn(III) is the rate-limiting step for the entire reaction. Soldatova *et al*.^3^ proposed that the MnxG-gene-product active site may be organized to couple successive electron transfers for the formation of polynuclear Mn(IV) complexes in this strain. In all of these cases, MCO is the key enzyme that directly catalyzes Mn(II) oxidation into Mn(III), whereas other proteins encoded in the same operon appear to be organized for the full oxidation of Mn(II) to Mn(IV). However, unlike other MCOs (e.g., MnxG and MofA) encoded in an operon comprising several genes, the MCO266 in MB266 is monocistronic. MCO266 also catalyzes only two single-electron transfer reactions and not the usual four electron transfers to O_2_^39^.

Therefore, we speculate that MCO266 catalyzes one-electron oxidation by converting Mn(II) into Mn(III) and then cooperates with other cellular membrane-bound electron transporters to perform successive electron transfers that oxidize Mn(III) to Mn(IV). The Mn(III) and Mn(IV) oxides produced by the recombinant *E. coli* strains with surface-displayed MCO266 (Fig. 6 and Table S1) may arise from an uncoordinated electron transfer from MCO266 to subsequent membrane electron transporters. Despite the instability of Mn(III) in its ionic state, it can form stable Mn(III) oxides in the presence of oxygen; this process is facilitated when Mn(III) is adsorbed onto a solid medium or combined with organic substrates to form chelating complexes^40^.

The formation of the aggregates appeared to require joint interactions between Mn oxides, MCO266, and other molecules attached to the bacterial cell surface. However, the MCO266 protein plays a key role in Mn oxidation and the successive formation of deposits and aggregates, as confirmed by the introduction of Mn(II)-oxidizing activity and aggregate formation into the activity-negative JM109 strain expressing MCO266 on the cell surface (Figs. 2,4). The data shown in Figs. 4 and S5 confirm that Mn(II) oxides contribute to aggregate formation. These results are consistent with those reported by Miller *et al*.^41^, who confirmed that naturally occurring Mn oxides bind to certain proximal proteins, microelements and microorganisms to form aggregates. Moreover, the self-aggregation of MCO266 proteins and the induction of aggregation by MCO266 should contribute to aggregate formation. Several previous investigations have confirmed that CotA laccase not only self-aggregates^42^,^43^ but also induces the conjugation between proteins and polysaccharides^44^. Thus, we propose that surface MCO266 not only directly oxidizes Mn(II) to form Mn oxides but also self-aggregates and induces multiple aggregations of proteins, polysaccharide macromolecules, and intact cells during aggregate biogenesis. During the continuous culturing process, the MCO266 proteins released from lytic cells can intensify the aggregations. Therefore, the strains expressing and/or displaying MCO266 on the cell surface initiate the formation of large, compact and multicomponent aggregates. However, disruption of the *mco266* gene in the genome of strain MB267, which resulted in fewer Mn oxides and the disappearance of MCO266 on the cell surfaces, correlated with the formation of smaller and incompact cell aggregates by MB267 after a continuous 14-d culturing process (Fig. 3).

The aggregates formed by the recombinant *E. coli* strains with surface-displayed MCO266 and the wild-type MB266 may initiate the formation of bulkier agglomerates through the participation of various types of ions and organic materials in the natural environment, ultimately forming Mn micronodules under the influence of pressure, temperature, and other factors. Based on the results obtained in this study, we propose the following outline for MCO-related Mn(II) oxidation and aggregate formation (Fig. S6): A two-component regulatory system (i.e., the Mnx system^45^) in the cells may trigger or accelerate oxidase expression and transmembrane transport when the environmental Mn(II) concentration reaches a certain threshold value. Prior to Mn(II) oxidation, Mn(II) may be combined with organic (i.e., oxalate, malonate, etc.) or inorganic (i.e., pyrophosphate) substrates to form Mn chelates^46^,^47^. The binding of Mn chelates to cell-surface-associated MCO266 activates the entire Mn(II) oxidation reaction. MCO266 catalyzes the conversion of Mn(II) into Mn(III), and Mn(III) is then transported to other redoxases (i.e., the CCM) to be oxidized into Mn(IV) in association with the reduction reaction of O_2_ to H_2_O. As the reaction proceeds, more Mn(III) and Mn(IV) oxides assemble on the cell surface, forming an Mn-oxide deposit layer. The layers on the cell surface may become gradually superimposed, resulting in various microspherical aggregates. These bacterial spheroids with high-potential Mn oxides may further serve as preliminary nucleation centers to oxidize and adsorb other metal ions or organic substrates, thereby successively forming bulkier aggregates. These aggregates can ultimately form ferromanganese or polymetallic micronodules under the influence of natural factors, such as pressure and temperature.

## Materials and Methods

### Plasmids, strains, culture conditions, and Mn(II)-oxidizing activity assay

Table S3 shows the strains, plasmids, and oligonucleotide primers used in this study. The wild-type *E. coli* MB266 was isolated from a surface soil with Fe−Mn nodules in Shangdong Province, China. The bacteria were cultured on Lept medium containing 20 mM 4-(2-hydroxyethyl)-1-piperazineethanesulfonic acid (HEPES, pH 7.2) and 1 mM Mn(II) at 30 °C^48^. The medium was supplemented with 100 μg ml^−1^ ampicillin (Amp) and 0.1 mM isopropyl-β-D-thiogalactoside (IPTG) once the culture OD_600_ of the *E. coli* constructs reached 0.6. For the production of Mn oxides, the overnight-cultured bacteria were inoculated into Lept medium at a ratio of 2% (V/V), and MnCl_2_ was added to the medium to achieve a final concentration of 1 mM. The cultures were cultivated continuously at 30 °C with shaking at 200 rpm for 5 d or 14 d, unless stated otherwise. The LBB method was used for Mn oxide quantification^49^,^50^ with the addition of 1.0 mM MnCl_2_ (final concentration) in Lept medium. The cells were cultured for 120 h at 37 °C unless otherwise stated. For the preparation of the heat-killed *E. coli* cell samples, the cells were incubated to an OD_600_ of 1.2, and the cultures were then shifted to a water bath at 65 °C for further incubation for 24 h. Twenty microliters of each treated cell suspension were inoculated into fresh LB broth to examine the lethal effect. Cell-free Lept medium was used in parallel as a control.

### Screening and identification of Mn(II)-oxidizing activity-deficient mutants

*E. coli* S17 λ-pir carrying pUTmini-Tn5 Km2 was used as the donor strain for the conjugative transfer of the mini-Tn5 transposon to *E. coli* MB266. Overnight cultures of S17 λ-pir and MB266 were resuspended in 10 mM MgSO_4_. Each culture containing 200 μl of S17 λ-pir (approximately 4.5 × 10^8^ CFUs ml^−1^) and 400 μl of MB266 (approximately 9 × 10^8^ CFUs ml^−1^) was plated and grown on an LB plate at 37 °C for 24 h. After conjugation, the cells were harvested and spread onto an LB plate containing 50 μg ml^−1^ of kanamycin (Kan) and 100 μg ml^−1^ of rifampicin to screen for resistant colonies. Each resistant colony was inoculated onto an LB plate containing 100 μg ml^−1^ Amp and 50 μg ml^−1^ Kan and an LB plate containing only 50 μg ml^−1^ Kan. The Amp-sensitive and Kan-resistant colonies were selected as the Tn5-inserted transconjugants (Kan^r^, Amp^s^). The Mn(II)-oxidizing activities of the transconjugants were then measured following the method described above. For inverse PCR, the total DNA of the transconjugants with little or no Mn(II)-oxidizing activity was extracted using a DNeasy DNA isolation kit (TaKaRa Bio, Inc., Dalian, China), digested with *Eco*R I and self-ligated using standard procedures^51^. The resultant self-ligated DNA samples were used as templates for inverse PCR in combination with the CKYP1 and CKYP2 primers, which are complementary to the internal sequence of mini-Tn5 for the first-round amplification. The amplified products were analyzed through agarose electrophoresis. The corresponding bands were incised, purified, and used as templates for the second-round PCR amplification with the CKJP1 and CKJP2 primers. The amplified products were sequenced using an ABI 373A automatic sequencer and were identified through a BLASTN search of the GenBank nucleotide sequence databases on the National Center for Biotechnology Information (NCBI) server (http://blast.ncbi.nlm.nih.gov/Blast.cgi).

### Cloning and disruption of the MB266 *mco266* gene

The *mco266* gene was PCR-amplified from the genome of MB266 using the mcoF_1_ and mcoR_1_ primers. The amplified products were subjected to sequencing. Disruption of the *mco266* gene in the MB266 genome was performed using the one-step chromosome gene inactivation method described by Baba *et al*.^28^.

### Plasmid construction

The *Bgl* II/*Eco*R I-digested PCR-amplified product from the *mco266* gene was ligated into the *Bgl* II/*Eco*R I sites of the *E. coli* expression vector pTrcHis B (Invitrogen, Carlsbad, CA92008, USA) to yield the pMB252 recombinant plasmid for the intracellular expression and *in vitro* purification of MCO266. A similarly digested fragment was inserted into the *Bgl* II/*Eco*R I sites of a previously constructed plasmid (pMB102)^29^ to yield the pMB253 recombinant plasmid expressing the InaQ-N/MCO266 fusion protein. Both the N- and C-terminal moieties of *mco266* (*mco266-N* and *mco266-C*) were amplified from the *mco266* gene using the mcoF_1_/mconR and mcocF/mcoR_1_ primers, respectively. The moieties were digested with *Bgl*II/*Eco*RI and inserted into the *Bgl*II/*Eco*RI sites of pMB102 to yield the pMB254 and pMB255 recombinant plasmids expressing the fusion proteins InaQ-N/MCO266-N and InaQ-N/MCO266-C, respectively.

### Expression, purification, protein cell-surface display, and cell fraction preparation

Protein expression in the recombinant *E. coli* strains was induced with 0.1 mM IPTG, and protein purification was performed using a His-tag purification system (Invitrogen) following the manufacturer’s instructions. To induce protein cell-surface display, the recombinant strains were induced with 0.1 mM IPTG. The surface localization of the corresponding proteins was detected by Western blot assays, immunofluorescence microscopy, and flow cytometry using polyclonal InaQ-N antiserum^52^ following previously described procedures^29^. Preparation of the COMC fraction was performed following previously described procedures^53^.

### TEM and SEM/EDX assays

Sample preparation, TEM, and SEM/EDX assays were performed following previously described procedures^19^,^54^.

### XPS analysis

The Mn(II)-oxidizing bacterial cultures were centrifuged and stored in a 20 mM HEPES buffer solution (pH 7.2). The suspensions were smashed six times at 15,000 psi using a French pressure cell (Thermo). Mn oxides were then collected by centrifugation at 5000 × g for 10 min and freeze-dried. XPS analysis of the obtained Mn oxides was conducted on a VG Multilab2000 X-ray photoelectron spectrometer with an Al Kα X-ray source (1486 eV) and a base pressure of 3 × 10^−9^ Torr in the analytical chamber^24^. The charge effect was corrected by adjusting the binding energy of adventitious C (1 s) to 284.62 eV. The Shirley-type background was subtracted before deconvolution and data fitting using the parameters used by Nesbitt *et al*.^55^ for the multiplet peaks of Mn (2p_3/2_) for spectra fitting. A 20 : 80 ratio of the Lorentzian–Gaussian mix-sum function was used for all data fittings^56^.

### XRD analysis

The culture products were freeze-dried to obtain biogenic manganese oxide for powder X-ray diffraction (XRD) analysis (Bruker D8 Advance X-ray diffractometer). The samples were placed on low-background quartz plates and scanned over the range 2θ = 5°– 85°. The measurement conditions included the following: a LynxEye array detector, a Ni filter plate, Cu Kα (λ = 0.15418 nm), a tube voltage of 40 kV, a tube current of 40 mA, step scan, a step size of 0.02°, and a scan speed of 10°/min.

## Additional Information

**How to cite this article**: Zhang, Z. *et al*. Surface Mn(II) oxidation actuated by a multicopper oxidase in a soil bacterium leads to the formation of manganese oxide minerals. *Sci. Rep*. **5**, 10895; doi: 10.1038/srep10895 (2015).

## Supplementary Material

Supplementary Information

## References

[b1] Tebo B. M., Johnson H. A., McCarthy J. K. & Templeton A. S. Geomicrobiology of manganese(II) oxidation. Trends Microbiol 13, 421−428 (2005). 16054815 10.1016/j.tim.2005.07.009

[b2] Tebo B. M. . Biogenic manganese oxides: Properties and mechanisms of formation. Annu Rev Earth Pl Sc 32, 287−328 (2004).

[b3] Soldatova A. V., Butterfield C., Oyerinde O. F., Tebo B. M. & Spiro T. G. Multicopper oxidase involvement in both Mn(II) and Mn(III) oxidation during bacterial formation of MnO_2_. J Biol Inorg Chem 17, 1151−1158 (2012). 22892957 10.1007/s00775-012-0928-6 PMC3743667

[b4] Clement B. G., Luther G. W. III & Tebo B. M. Rapid, oxygen-dependent microbial Mn(II) oxidation kinetics at sub-micromolar oxygen concentrations in the Black Sea suboxic zone. Geochim Cosmochim Ac 73, 1878−1889 (2009).

[b5] Dick G. J. . Enzymatic microbial Mn(II) oxidation and Mn biooxide production in the Guaymas Basin deep-sea hydrothermal plume. Geochim Cosmochim Ac 73, 6517−6530 (2009).

[b6] Zhang J. H., Lion L. W., Nelson Y. M., Shuler M. L. & Ghiorse W. C. Kinetics of Mn(II) oxidation by *Leptothrix discophora* SS1. Geochim Cosmochim Ac 66, 773−781 (2002).

[b7] Benner S. G., Blowes D. W., Gould W. D., Herbert R. B. Jr. & Ptacek C. J. Geochemistry of a permeable reactive barrier for metals and acid mine drainage. Environ Sci Technol 33, 2793–2799 (1999).

[b8] Jambor J. L., Raudsepp M. & Mountjoy K. Mineralogy of permeable reactive barriers for the attenuationof subsurface contaminants. Can Mineral 43, 2117–2140 (2005).

[b9] Lee J. H. . Manganese sulfide formation via concomitant microbial manganese oxide and thiosulfate reduction. Environ Microbiol 13, 3275−3288 (2011). 21951417 10.1111/j.1462-2920.2011.02587.x

[b10] Corstjens P. L. A. M., de Vrind J. P. M., Goosen T. & Jong E. W. d. V. d. Identification and molecular analysis of the *Leptothrix discophora* SS‐1 *mofA* gene, a gene putatively encoding a manganese—oxidizing protein with copper domains. Geomicrobiol J 14, 91−108 (1997).

[b11] Caspi R., Tebo B. M. & Haygood M. G. *c*-type cytochromes and manganese oxidation in *Pseudomonas putida* MnB1. Appl Environ Microbiol 64, 3549−3555 (1998). 9758766 10.1128/aem.64.10.3549-3555.1998 PMC106463

[b12] Brouwers G. J. . *cumA*, a gene encoding a multicopper oxidase, is involved in Mn^2+^ oxidation in *Pseudomonas putida* GB-1. Appl Environ Microbiol 65, 1762−1768 (1999). 10103278 10.1128/aem.65.4.1762-1768.1999 PMC91248

[b13] van Waasbergen L. G., Hoch J. A. & Tebo B. M. Genetic analysis of the marine manganese-oxidizing *Bacillus* sp. strain SG-1: protoplast transformation, Tn917 mutagenesis, and identification of chromosomal loci involved in manganese oxidation. J Bacteriol 175, 7594−7603 (1993). 8244928 10.1128/jb.175.23.7594-7603.1993 PMC206915

[b14] van Waasbergen L. G., Hildebrand M. & Tebo B. M. Identification and characterization of a gene cluster involved in manganese oxidation by spores of the marine *Bacillus* sp. strain SG-1. J Bacteriol 178, 3517−3530 (1996). 8655549 10.1128/jb.178.12.3517-3530.1996 PMC178121

[b15] Sullivan L. & Koppi A. Manganese oxide accumulations associated with some soil structural pores.II. Composite coatings and translocation. Soil Res 31, 227−233 (1993).

[b16] He J., Zhang L., Jin S., Zhu Y. & Liu F. Bacterial communities inside and surrounding soil iron-manganese nodules. Geomicrobiol J 25, 14−24 (2008).

[b17] Ridge J. P. . A multicopper oxidase is essential for manganese oxidation and laccase-like activity in *Pedomicrobium* sp. ACM 3067. Environ Microbiol 9, 944−953 (2007). 17359266 10.1111/j.1462-2920.2006.01216.x

[b18] Brouwers G. J. . Stimulation of Mn^2+^ Oxidation in *Leptothrix discophora* SS-1 by Cu^2+^ and sequence analysis of the region flanking the gene encoding putative multicopper oxidase MofA. Geomicrobiol J 17, 25−33 (2000).

[b19] Francis C. A., Casciotti K. L. & Tebo B. M. Localization of Mn(II)-oxidizing activity and the putative multicopper oxidase, MnxG, to the exosporium of the marine *Bacillus* sp. strain SG-1. Arch Microbiol 178, 450−456 (2002). 12420165 10.1007/s00203-002-0472-9

[b20] Kataoka K. . Structure and function of the engineered multicopper oxidase CueO from *Escherichia coli*—deletion of the methionine-rich helical region covering the substrate-binding site. J Mol Biol 373, 141−152 (2007). 17804014 10.1016/j.jmb.2007.07.041

[b21] Butterfield C. N., Soldatova A. V., Lee S. W., Spiro T. G. & Tebo B. M. Mn(II,III) oxidation and MnO_2_ mineralization by an expressed bacterial multicopper oxidase. Proc Natl Acad Sci USA 110, 11731−11735 (2013). 23818588 10.1073/pnas.1303677110 PMC3718108

[b22] Taylor R. J., Reid M., Foot J. & Raw S. A. Tandem oxidation processes using manganese dioxide: discovery, applications, and current studies. Acc Chem Res 38, 851−869 (2005). 16285708 10.1021/ar050113t

[b23] Huston W. M., Jennings M. P. & McEwan A. G. The multicopper oxidase of *Pseudomonas aeruginosa* is a ferroxidase with a central role in iron acquisition. Mol Microbiol 45, 1741−1750 (2002). 12354238 10.1046/j.1365-2958.2002.03132.x

[b24] Singh S. K., Grass G., Rensing C. & Montfort W. R. Cuprous oxidase activity of CueO from Escherichia coli. J Bacteriol 186, 7815−7817 (2004). 10.1128/JB.186.22.7815-7817.2004 PMC524913 15516598

[b25] Solomon E. I., Sundaram U. M. & Machonkin T. E. Multicopper oxidases and oxygenases. Chem Rev 96, 2563−2606 (1996). 11848837 10.1021/cr950046o

[b26] Roberts S. A. . A labile regulatory copper ion lies near the T1 copper site in the multicopper oxidase CueO. J Biol Chem 278, 31958−31963 (2003). 12794077 10.1074/jbc.M302963200

[b27] Cui X. Y. . Isolation and identification of an *Escherichia coli* strain with high manganese-oxidizing activity and cloning and characterization of its multicopper oxidase gene. Microbiol Chn 38, 1465−1472 (2011).

[b28] Baba T. . Construction of *Escherichia coli* K-12 in-frame, single-gene knockout mutants: the Keio collection. Mol Syst Biol 2, 2006.0008 (2006). 10.1038/msb4100050 PMC1681482 16738554

[b29] Li Q., Yu Z., Shao X., He J. & Li L. Improved phosphate biosorption by bacterial surface display of phosphate-binding protein utilizing ice nucleation protein. FEMS Microbiol Lett 299, 44−52 (2009). 19686343 10.1111/j.1574-6968.2009.01724.x

[b30] de Silva D., Davis-Kaplan S., Fergestad J. & Kaplan J. Purification and characterization of Fet3 protein, a yeast homologue of ceruloplasmin. J Biol Chem 272, 14208−14213 (1997). 9162052 10.1074/jbc.272.22.14208

[b31] Hua M. . Heavy metal removal from water/wastewater by nanosized metal oxides: a review. J Hazard Mater 211-212, 317−331 (2012). 22018872 10.1016/j.jhazmat.2011.10.016

[b32] Xu W., Wang H., Liu R., Zhao X. & Qu J. The mechanism of antimony(III) removal and its reactions on the surfaces of Fe-Mn binary oxide. J Colloid Interface Sci 363, 320−326 (2011). 21840528 10.1016/j.jcis.2011.07.026

[b33] Yin H., Feng X., Qiu G., Tan W. & Liu F. Characterization of Co-doped birnessites and application for removal of lead and arsenite. J Hazard Mater 188, 341−349 (2011). 21345580 10.1016/j.jhazmat.2011.01.129

[b34] Morgan J. J. Kinetics of reaction between O_2_ and Mn(II) species in aqueous solutions. Geochim Cosmochim Ac 69, 35−48 (2005).

[b35] Ren H. T. . Abiotic oxidation of Mn(II) induced oxidation and mobilization of As(III) in the presence of magnetite and hematite. J Hazard Mater 254-255, 89−97 (2013). 23587932 10.1016/j.jhazmat.2013.03.022

[b36] Farnsworth C. E., Voegelin A. & Hering J. G. Manganese oxidation induced by water table fluctuations in a sand column. Environ Sci Technol 46, 277−284 (2012). 22126514 10.1021/es2027828

[b37] Nakamura K. & Go N. Function and molecular evolution of multicopper blue proteins. Cell Mol Life Sci 62, 2050−2066 (2005). 16091847 10.1007/s00018-004-5076-x PMC11139126

[b38] Webb S. M., Dick G. J., Bargar J. R. & Tebo B. M. Evidence for the presence of Mn(III) intermediates in the bacterial oxidation of Mn(II). Proc Natl Acad Sci USA 102, 5558−5563 (2005). 15800042 10.1073/pnas.0409119102 PMC556228

[b39] Giardina P. . Laccases: a never-ending story. Cell Mol Life Sci 67, 369−385 (2010). 19844659 10.1007/s00018-009-0169-1 PMC11115910

[b40] Liu G. F. . Chelate electronic properties control the redox behaviour and superoxide reactivity of seven-coordinate manganese(II) complexes. Dalton Trans, 6292−6295 (2009). 10.1039/b906100m. 19655061

[b41] Miller A. Z. . Biogenic Mn oxide minerals coating in a subsurface granite environment. Chem Geol 322–323, 181−191 (2012).

[b42] Fernandes A. T., Lopes C., Martins L. O. & Melo E. P. Unfolding pathway of CotA-laccase and the role of copper on the prevention of refolding through aggregation of the unfolded state. Biochem Biophys Res Commun 422, 442−446 (2012). 22579799 10.1016/j.bbrc.2012.05.011

[b43] Shleev S. . Autoreduction and aggregation of fungal laccase in solution phase: possible correlation with a resting form of laccase. Biochimie 88, 1275−1285 (2006). 16581176 10.1016/j.biochi.2006.02.007

[b44] Gazme B. & Madadlou A. Fabrication of whey protein–pectin conjugate particles through laccase-induced gelation of microemulsified nanodroplets. Food Hydrocolloid 40, 189−195 (2014).

[b45] Geszvain K. & Tebo B. M. Identification of a two-component regulatory pathway essential for Mn(II) oxidation in *Pseudomonas putida* GB-1. Appl Environ Microbiol 76, 1224−1231 (2010). 20038702 10.1128/AEM.02473-09 PMC2820985

[b46] Hofer C. & Schlosser D. Novel enzymatic oxidation of Mn^2+^ to Mn^3+^ catalyzed by a fungal laccase. FEBS Lett 451, 186−190 (1999). 10371162 10.1016/s0014-5793(99)00566-9

[b47] Schlosser D. & Hofer C. Laccase-catalyzed oxidation of Mn^2+^ in the presence of natural Mn^3+^ chelators as a novel source of extracellular H_2_O_2_ production and its impact on manganese peroxidase. Appl Environ Microbiol 68, 3514−3521 (2002). 12089036 10.1128/AEM.68.7.3514-3521.2002 PMC126759

[b48] Boogerd F. C. & de Vrind J. P. Manganese oxidation by *Leptothrix discophora*. J Bacteriol 169, 489−494 (1987). 3804969 10.1128/jb.169.2.489-494.1987 PMC211803

[b49] Johnson H. A. & Tebo B. M. *In vitro* studies indicate a quinone is involved in bacterial Mn(II) oxidation. Arch Microbiol 189, 59−69 (2008). 17673976 10.1007/s00203-007-0293-y PMC2721854

[b50] Krumbein W. E. & Altmann H. J. A new method for the detection and enumeration of manganese oxidizing and reducing microorganisms. Helgol wiss Meeresunters 25, 347−356 (1973).

[b51] Sambrook. J. & Russell. D. W. Molecular cloning: A laboratory manual. (Cold Spring Harbor Laboratory Press, 2001).

[b52] Li Q. . Suppressing *Erwinia carotovora* pathogenicity by projecting *N*-acyl homoserine lactonase onto the surface of *Pseudomonas putida* cells. J Microbiol Biotechnol 21, 1330−1335 (2011). 22210621 10.4014/jmb.1107.07011

[b53] Li L., Kang D. G. & Cha H. J. Functional display of foreign protein on surface of *Escherichia coli* using N-terminal domain of ice nucleation protein. Biotechnol Bioeng 85, 214−221 (2004). 14705004 10.1002/bit.10892

[b54] Michalak I., Chojnacka K. & Marycz K. Using ICP-OES and SEM-EDX in biosorption studies. Mikrochim Acta 172, 65−74 (2011). 21423317 10.1007/s00604-010-0468-0 PMC3029800

[b55] Nesbitt H. W., Canning, G. W. & Bancroft, G. M. XPS study of reductive dissolution of 7Å-birnessite by H_3_AsO_3_, with constraints on reaction mechanism. Geochim Cosmochim Ac 62, 2097−2110 (1998).

[b56] Yin H. . Co^2+^-exchange mechanism of birnessite and its application for the removal of Pb^2+^ and As(III). J Hazard Mater 196, 318−326 (2011). 21963172 10.1016/j.jhazmat.2011.09.027

