# Supplementary material for: Surface Mn(II) oxidation actuated by a multicopper oxidase in a soil bacterium leads to the formation of manganese oxide minerals
Source: Sci Rep. 2015 Jun 3;5:10895. doi: 10.1038/srep10895 (PMC4454072; doi:10.1038/srep10895)
Supplement: Supplementary Information [file srep10895-s1.pdf]

Surface Mn(II) oxidation actuated by a multicopper oxidase in a soil bacterium leads to the formation of manganese oxide minerals

Zhen Zhang[1,2], Zhongming Zhang[1], Hong Chen[1], Jin Liu[1], Chang Liu[1], Hong Ni[3], Changsong Zhao[2], Muhammad Ali[1], Fan Liu[2], Lin Li[1,*]

[1] *State Key Laboratory of Agricultural Microbiology, Huazhong Agricultural University, Wuhan 430070, China*

[2] *Key Laboratory of Subtropical Agricultural Resource and Environment, Ministry of Agriculture, Huazhong Agricultural University, Wuhan 430070, China*

[3]*School of Life Science, Hubei University, Wuhan 430062, China*

* Corresponding author. State Key Laboratory of Agricultural Microbiology, Huazhong Agricultural University, Wuhan, Hubei Province, China. Tel: +86-027-87286952; E-mail: lilin@mail.hzau.edu.cn.

Supplementary Information

1. Supplementary Tables

Table S1. **Mn ($2p_{3/2}$) peak parameters for MB266, MB261 and MB253 Mn oxides**

Peak	B.E (ev)	FWHM (ev)	Percent (%) MB266	MB261	MB253	Surface species
**Mn^{2+}($2p_{3/2}$) parameters**			Mn^{2+} (total) At. %			
			33.68 ± 0.1	31.74 ± 0.1	24.57 ± 0.1	
Mn^{2+}	639.75	1.15	11.42	14.44	5.60	Mn(II)-O Multiplet #1
Mn^{2+}	640.95	1.15	10.93	7.53	5.83	Mn(II)-O Multiplet #2
Mn^{2+}	641.75	1.15	6.56	5.30	5.52	Mn(II)-O Multiplet #3
Mn^{2+}	642.65	1.15	2.75	2.63	3.69	Mn(II)-O Multiplet #4
Mn^{2+}	644.15	1.15	2.02	1.84	3.93	Mn(II)-O Multiplet #5
**Mn^{3+}($2p_{3/2}$) parameters**			Mn^{3+} (total) At. %			
			34.02 ± 0.1	34.73 ± 0.1	52.8 ± 0.1	
Mn^{3+}	640.65	1.15	16.7	15.26	22.51	Mn(III)-O Multiplet #1
Mn^{3+}	641.35	1.15	9.43	8.28	17.26	Mn(III)-O Multiplet #2
Mn^{3+}	642.16	1.15	6.68	7.51	9.20	Mn(III)-O Multiplet #3
Mn^{3+}	643.18	1.15	0	2.68	3.61	Mn(III)-O Multiplet #4
Mn^{3+}	644.55	1.15	1.21	1.00	0.22	Mn(III)-O Multiplet #5
**Mn^{4+}($2p_{3/2}$) parameters**			Mn^{4+} (total) At. %			
			32.29 ± 0.1	33.51 ± 0.1	22.63 ± 0.1	
Mn^{4+}	641.90	1.15	14.87	9.15	14.63	Mn(IV)-O Multiplet #1
Mn^{4+}	642.92	1.15	9.86	7.33	1.41	Mn(IV)-O Multiplet #2
Mn^{4+}	643.75	1.15	3.85	7.66	0.27	Mn(IV)-O Multiplet #3
Mn^{4+}	644.78	1.15	3.40	7.54	3.78	Mn(IV)-O Multiplet #4
Mn^{4+}	645.80	1.15	0.31	1.83	2.54	Mn(IV)-O Multiplet #5

Table S2. Effects of various chemical components on the Mn(II)-oxidizing activity levels of *E. coli* MB266 and MB253

Compound [a]		Concentration of Mn oxides formed by MB266 (μM) (means ± SD [b], n = 4)	Concentration of Mn oxides formed by MB253 (μM) (means ± SD, n = 4)
Sodium azide	1 mM	20 ± 4	9 ± 2
	10 mM	12 ± 2	3 ± 1
SDS	1 mM	53 ± 18	83 ± 36
	10 mM	38 ± 16	73 ± 29
Cytochrome c	10 μM	60 ± 9	95 ± 7
	100 μM	44 ± 5	68 ± 5
ATP	200 μM	68 ± 9	123 ± 17
	2000 μM	49 ± 7	96 ± 9
PQQ	10 μM	88 ± 10	176 ± 23
	100 μM	99 ± 11	204 ± 28
NADH	20 μM	82 ± 13	169 ± 12
	200 μM	86 ± 15	198 ± 21
NAD^+	20 μM	77 ± 9	171 ± 14
	200 μM	81 ± 11	183 ± 0
Sodium oxalate	1 mM	77 ± 10	168 ± 20
	10 mM	86 ± 14	184 ± 21
Control		65 ± 8	125 ± 16

[a] Abbreviates: SDS, sodium dodecyl sulfate; ATP, adenosine 5′-triphosphate disodium salt hydrate; PQQ, pyrroloquinoline quinine; NAD^+, β-nicotinamide adenine dinucleotide sodium salt; NADH, β-nicotinamide adenine dinucleotide, reduced disodium salt hydrate.

[b] SD, standard deviation.

Table S3. Plasmids, strains and oligonucleotide primers used in this study

Plasmids, stains or primers	Phenotypes or sequences [a]	Sources or references
Plasmids		
pTrcHis B	Amp^r; *E. coli* expression vector, 4412 bps	Invitrogen
pTrcHis C	Amp^r; *E. coli* expression vector, 4412 bps	Invitrogen
pMB102	Amp^r, pTrcHis-C derivative harboring P_{trc} and fusion gene *inaQ-N*/*gfp*, 5543 bp	Li et al.[1]
pMB252	Amp^r, pTrcHis-B derivative harboring P_{trc} and *mco*, 5937 bp	This study
pMB253	Amp^r, pMB102 derivative harboring fusion gene *inaQ-N/mco*, 6358 bp	This study
pMB254	Amp^r, pMB102 derivative harboring fusion gene *inaQ-N/mco-N*, 5860 bp	This study
pMB255	Amp^r, pMB102 derivative harboring fusion gene *inaQ-N/mco-C*, 5362 bp	This study
pMB257	Amp^r, pTrcHis-C derivative harboring P_{trc} and *ccmF, ccmG, ccmH* and *mco* gene, 9389 bp	This study
pMB258	Amp^r, pTrcHis-C derivative harboring P_{trc} and *ccmF*, 6342 bp	This study
pMB259	Amp^r, pTrcHis-C derivative harboring P_{trc} and *ccmG*, 4956 bp	This study
pMB260	Amp^r, pTrcHis-C derivative harboring P_{trc} and *ccmH*, 5451 bp	This study
Escherichia coli strains		
MB266	Amp^sKan^s, Wide-type strain with high Mn(II)-oxidizing activity	This study
S17 λ-pir	Carrying conjugation-transfer plasmid pUT mini-Tn5 KM2	Biomedal, Seville, Spain
JM109	*recA1*Δ*(lac-proAB)* /F' *relA*1 *endA*1 *gyrA*96 *thi hsdR*17 *sup E*44	Laboratory collection

MB252	Amp^r, transformed *E. coli* JM109 harboring pMB252	This study
MB253	Amp^r, transformed *E. coli* JM109 harboring pMB253	This study
MB254	Amp^r, transformed *E. coli* JM109 harboring pMB254	This study
MB255	Amp^r, transformed *E. coli* JM109 harboring pMB255	This study
MB261	Amp^r, transformed *E. coli* MB266 harboring pMB253	This study
MB267	Amp^s, MB266 derivative with disrupted *mco* gene	This study
MB268	Amp^r, transformed *E. coli* MB267 harboring pMB252	This study
MB271	Amp^r, transformed *E. coli* JM109 harboring pMB257	This study
MB272	Amp^r, transformed *E. coli* JM109 harboring pMB258	This study
MB273	Amp^r, transformed *E. coli* JM109 harboring pMB259	This study
MB274	Amp^r, transformed *E. coli* JM109 harboring pMB260	This study
Oligonucleotide primers [b]		
mcoF1	5′–CGAAGATCTATGCAACGTCGTGATTTC–3′ (*Bgl*II)	
mcoR1	5′–ACGGAATTCTTATACCGTAAACCCTAAC–3′ (*Eco*RI)	
mocnR	5′–TTCGAATTCAGGTAACGCAGGCAGGCT–3′ (*Eco*RI)	
mcocF	5′–GCTAGATCTTCGCTGGAAGGGCTGAC–3′ (*Bgl*II)	
CKYP1	5′–GCGCAGGGCTTTATTGATTC–3′	
CKYP2	5′–GCTATTCTGACCTTGCCATC–3′	
CKJP1	5′–CTCTAGAGTCGACCTGCAGG–3′	
CKJP2	5′–CACTGATGAATGTTCCGTTGC–3′	

[a] Amp^r/Amp^s, ampicillin resistance/sensitivity; Kan^s*, kanamycin* sensitive; P_{trc}, promoter of *trc*; *inaQ-N,* N-terminal domain of *inaQ*; *gfp*, green fluorescent protein gene; *mco*, multicopper oxidase gene from MB266; *mco-N*, N-terminal domain of *mco*; *mco-C*, C-terminal domain of *mco*; *ccmFGH*, F, G and H domains of *c*-cytochrome mature operon in MB266, respectively.

[b] The underlined sequences are the restriction enzyme sites.

References

1. Li, Q., Yu, Z., Shao, X., He, J. & Li, L. Improved phosphate biosorption by bacterial surface display of phosphate-binding protein utilizing ice nucleation protein. *FEMS Microbiol Lett* **299**, 44–52.

2. Supplementary Figures

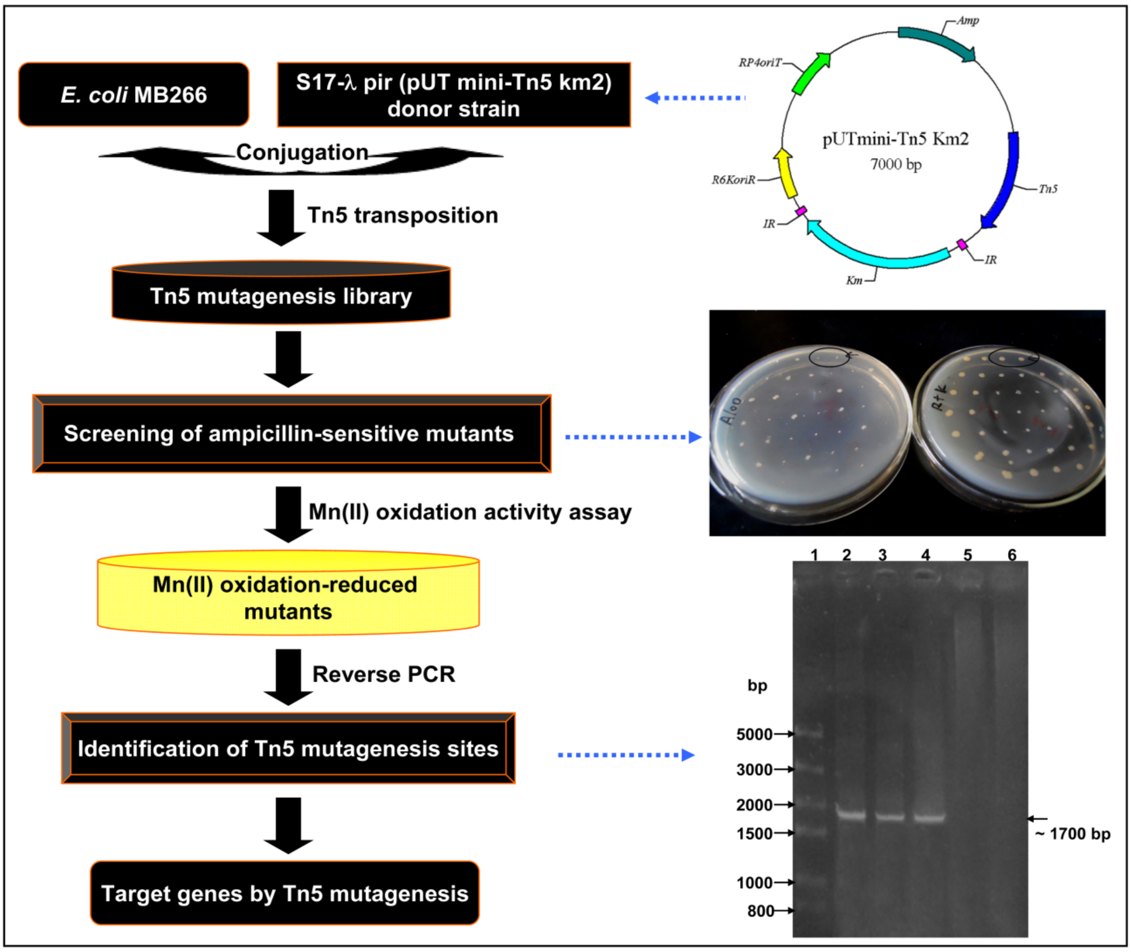

Figure S1. Schematic illustration of Tn5 mutagenesis in the *E. coli* wild-type strain MB266 and identification of the Tn5 mutagenesis sites and target genes.

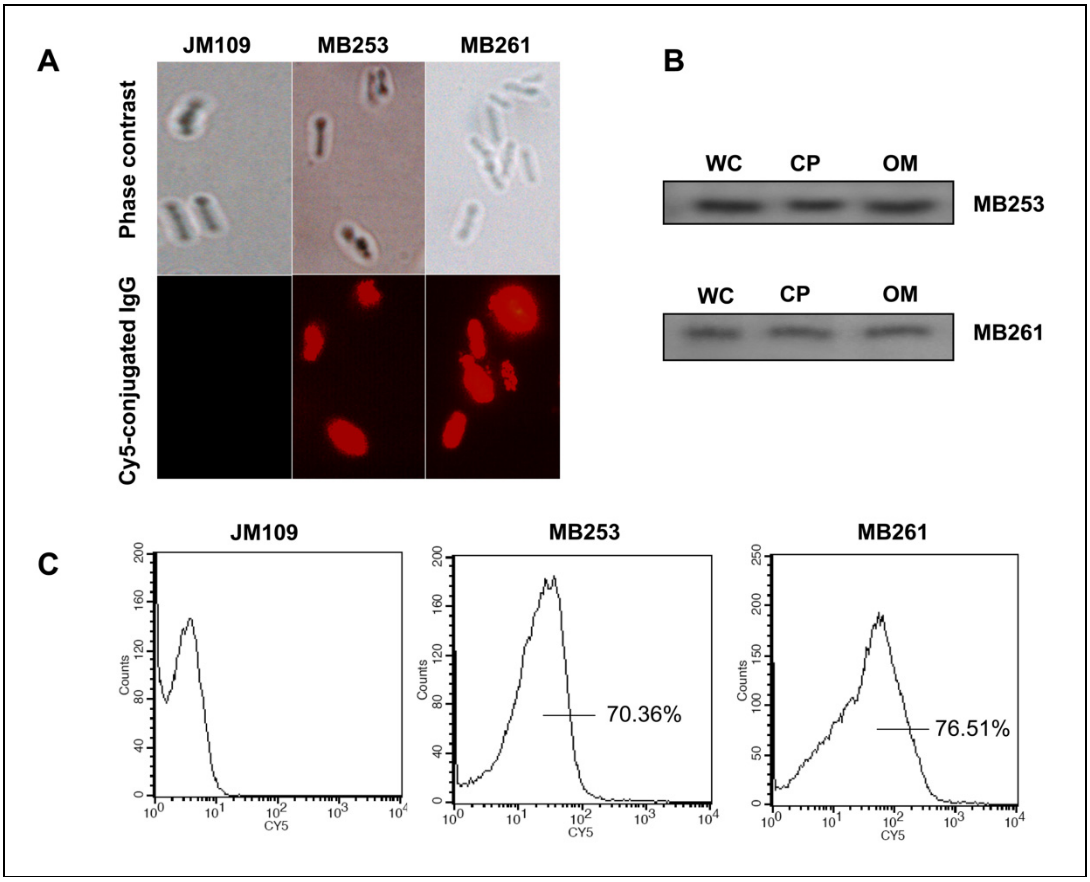

Figure S2. **Surface localization analysis of immobilized InaQ-N/MCO266 in recombinant *E. coli* MB253 and MB261 cells.** A: Immunofluorescence microscopy observation of intact cells. B: Western blot analysis of the cell fractions. WC, whole-cell fraction; CP, cell cytoplasmic fraction; OM, cell outer membrane fraction. C: Flow cytometric analysis of intact cells. The *E. coli* recipient strain JM109 was used as a control.

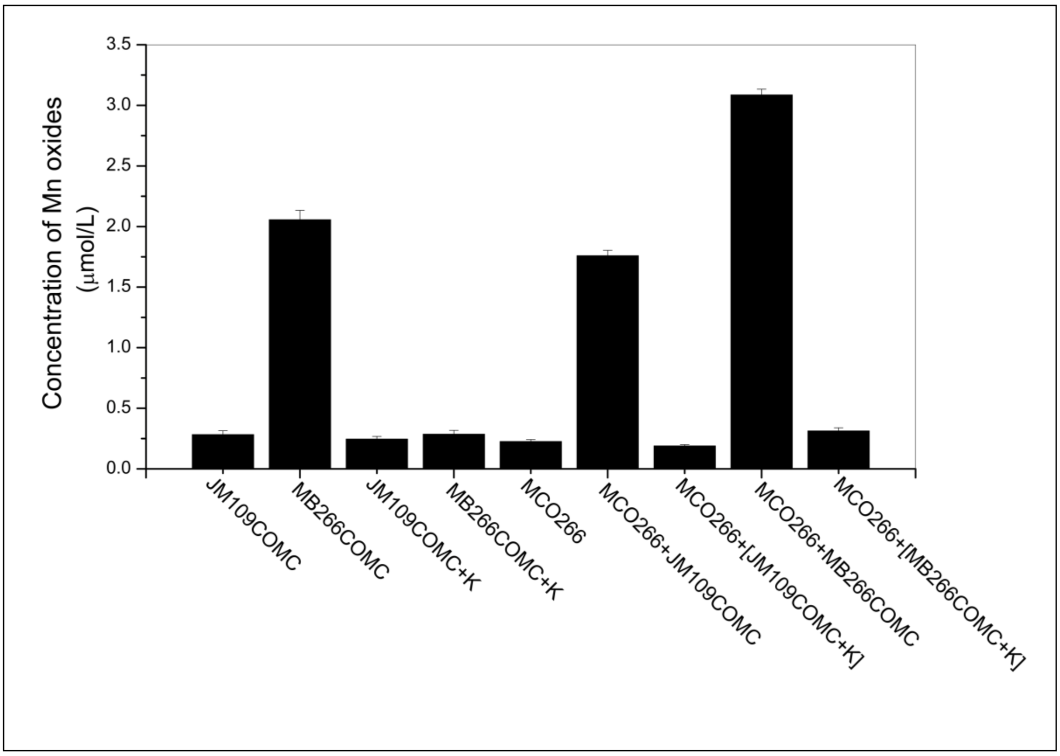

Figure S3. Determination of the Mn(II)-oxidizing activity of the mixtures with purified MCO266 and various purified COMC fractions with or without proteinase K proteolysis.

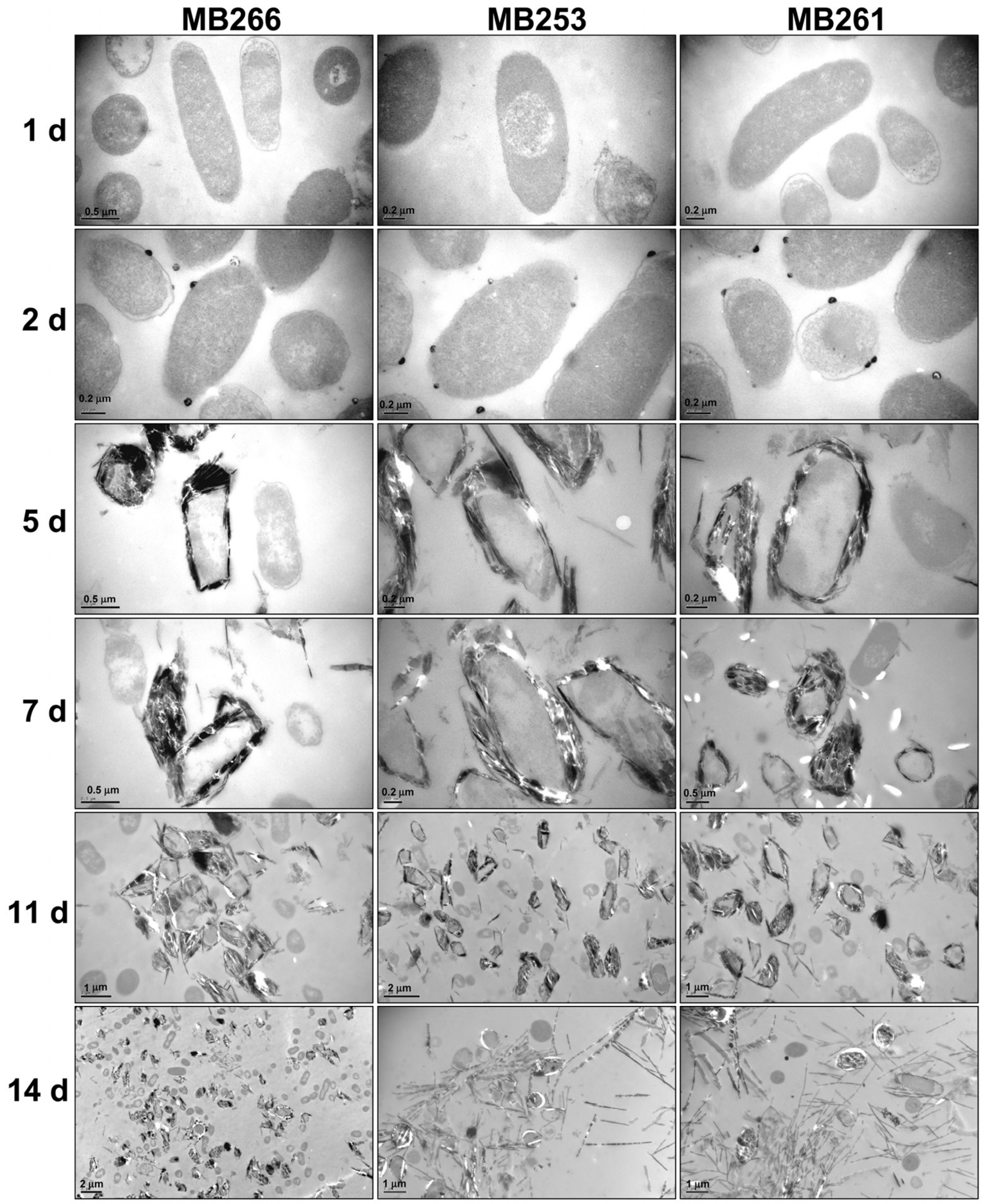

Figure S4. TEM micrographs of *E. coli* MB266, MB253 and MB261 over a period of 14 d. The cells were grown in liquid Lept medium in the presence of 1 mM Mn(II).

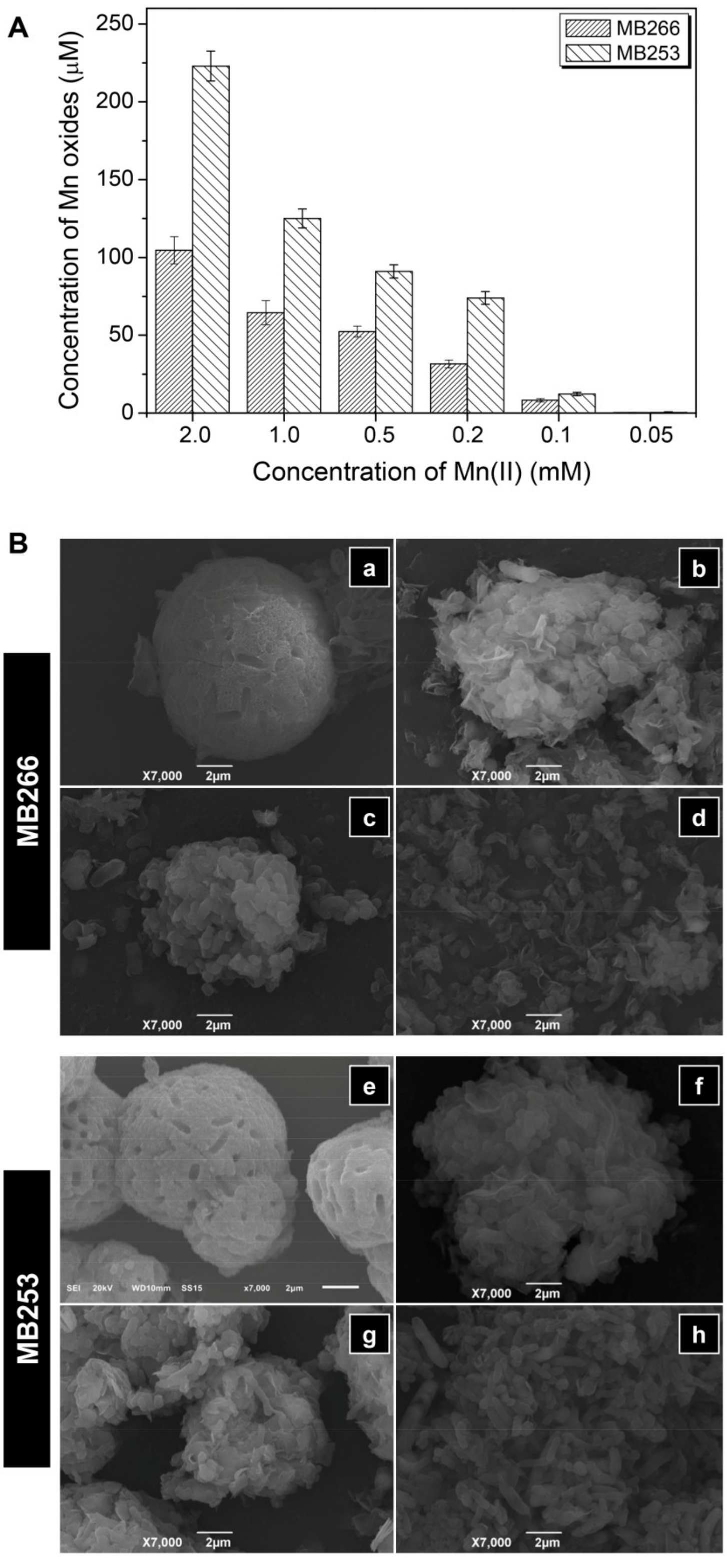

Figure S5. Effects of Mn(II) concentrations on the Mn(II)-oxidizing activity (A) and aggregate formation (B) of MB266 and MB253. A: The Mn(II)-oxidizing

activity was measured after culturing the cells for 120 h. B: MB266 and MB253 cells were cultured for 14 d. $MnCl_2$ concentrations: 1.0 mM (a; e); 0.5 mM (b; f); 0.2 mM (c; g); and 0.1 mM (d; h).

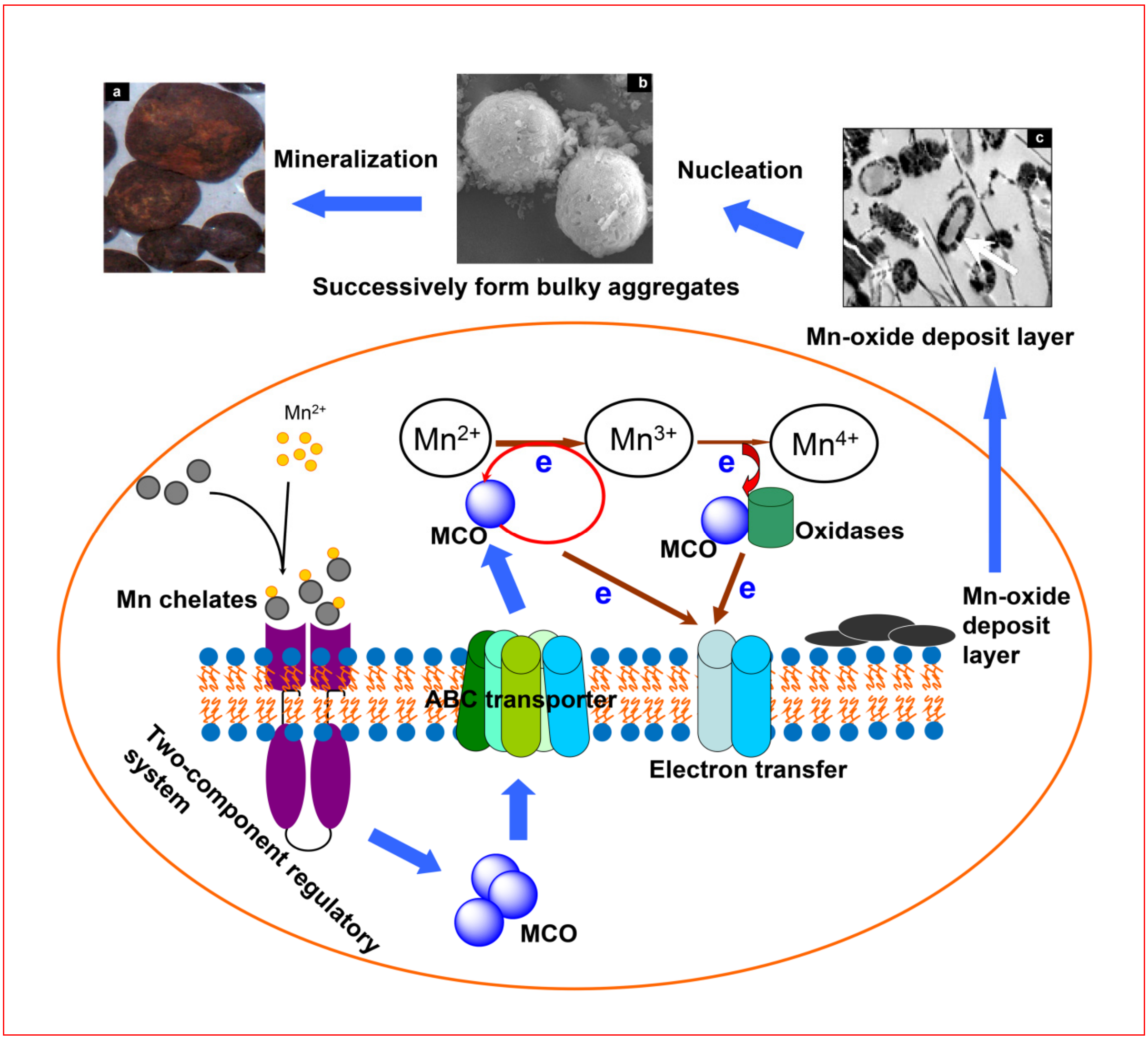

Figure S6. Presumed molecular mechanisms for Mn(II) oxidation and successive aggregate formation of Mn oxides in the *E. coli* MB266 strain. A: Fe–Mn nodules isolated from a brown soil sample harboring the *E. coli* MB266 strain. The sample was collected from Queyu, Shandong Province, China, by the authors in 2012. B: SEM micrograph of the microspherical aggregates formed by *E. coli* MB266 under laboratory Mn(II)-enriched culture conditions. C: TEM micrograph of *E. coli* MB266 cells under Mn(II)-enriched culture conditions.
